# At the intersection of machine learning, biology, and health: an interview with Lorin Crawford

**DOI:** 10.1038/s42003-020-01503-1

**Published:** 2021-01-04

## Abstract

Lorin Crawford began his independent career at Brown University School of Public Health with his own lab in the summer of 2017. He is currently a Senior Researcher at Microsoft Research, New England while also keeping his faculty position at Brown University. In this short Q&A he tells us about his research and the effect the pandemic has had on his lab and science. Dr. Crawford also shares some great tips on academic careers and making biostatistics approachable to wider audience and his views on the most exciting application of machine learning.

**Figure:**
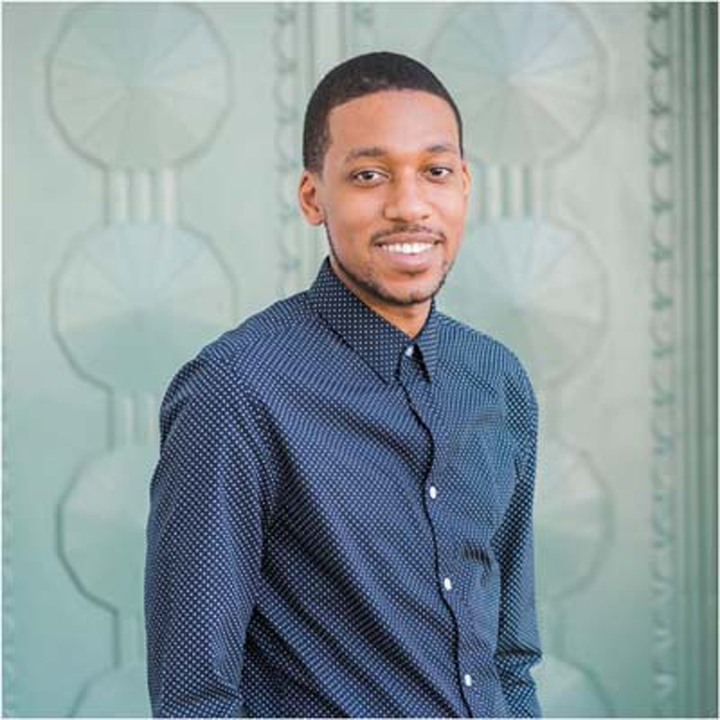
Elizabeth Burgi

Can you tell us about your research interests?

Yes, of course. The central theme to my research program is to develop statistical and machine learning algorithms that aid in the understanding of how relationships between genetic features affect the architecture of complex traits and contribute to disease etiology. I primarily work on problems in three different areas. The first is in statistical genetics, where I am interested in leveraging the nonlinear processing power of methods like neural networks and variational autoencoders to better characterize how interactions between genetic variants define the broad-sense heritability of traits like height and body mass index in human beings. I also think about a similar problem in cancer genomics, where my collaborators and I work to build algorithms that define rigorous transcriptional signatures of oncogenic events such as cancer recurrence and therapeutic resistance. Lastly, and most recently, I have been really interested in investigating when shape variation can be used to explain genotypic and phenotypic variation. Here, my ultimate goal is to build computational pipelines for analyzing 3D clinical imaging of soft tissues with the immediate plan to discover associations between tumor shapes and molecular properties like signaling pathway behavior.

Where did you find your initial motivation to pursue biostatistics as a career?

My journey to biostatistics was mainly motivated by my desire to do interdisciplinary research. I remember during my sophomore year as an undergraduate at Clark Atlanta University, I took two different courses in differential equations and statistics. I absolutely loved statistics. However, at the end of the differential equations course, we did a project where we were given the task to model the mutation rates of different immunodeficiency viruses. That was the first time that I had encountered a real-life example of mathematics being used at the intersection of biology to solve a significant problem in public health. It was after that school year that I sought out more opportunities that allowed me to investigate biostatistical type research questions.

Any tips on how to make biostatistics more approachable to students or a broader audience?

That’s a great question. What is beautiful about statistics is that many fundamental concepts within the field occur in many areas of our everyday lives. My goal in any presentation that I give is to have the audience walk away having learned at least three things. So, whenever I am teaching or giving talks, I like to relate seemingly complex topics to examples in pop culture or sports (or any topic that a general audience can relate to and find tangible). I also put a lot of thought into data visualization, and I tend to represent statistical models with imagery first and equations second. I think that helps people better connect with the meaning behind the math (even without any formal training).

How has the shutdown caused by the current pandemic affected your lab? Did it have any effect on student funding and career development?

Another great question. First, I want to acknowledge that we are very fortunate to be a computational or “dry” lab. So, from a research perspective, we have not been physically hindered from continuing to run numerical experiments, conducting analyses, and writing papers. However, in the bigger and more important picture, a lot of us have faced other challenges adjusting to this “new normal”. As a junior PI, I admit that it took me a while to find ways to be an effective mentor to students from afar—and I am still continuously learning new ways to improve upon that every day. With everything going on, both with the global pandemic and the heightened social unrest happening across America, I just try to make sure that I practice compassion and understanding for everyone’s individual situations. I also really encourage students in my group to make sure that they put their physical and mental health first.

Overall, it was a rough start in the beginning, but we have really started to hit our stride in the last couple of months as people become more comfortable and as we create a better virtual atmosphere. I also want to say that the sense of community that I have felt both at Brown and Microsoft has been amazing. Personally, I have been relieved that students in my group are funded by external grants and fellowships—but even then, I still have colleagues that routinely check in and make sure that the students I work with have the financial resources that they need to succeed. That includes making sure that they can attend virtual conferences or participate in online training webinars. I know that online career development is not ideal, but I have been encouraged by the collective effort in making sure that students and trainees are reaching the milestones that they want to meet.

What is the secret behind securing a tenure-track job, which in your case was right after completing a PhD? Do you have any tips for early career researchers?

Similar to my answer for the previous question: it takes a village and for me that village was Duke University. I had two amazing dissertation advisors during my PhD: Sayan Mukherjee (Statistical Science) and Kris Wood (Pharmacology and Cancer Biology). They somewhat treated me like a postdoctoral fellow and allowed me to develop and lead my own research projects. I think that level of academic freedom really gave me confidence as a young researcher to pursue the science that I found interesting. I absolutely would not be here without them. So, my first tip for early career researchers is to take choosing an advisor seriously, and really weigh the pros and cons of both working style and personality fit. I am huge believer that a great mentor can help a trainee maximize their ceiling.

I think the secret behind me securing a tenure-track job really boils down to a few factors. First, faculty within my department at Duke told me that I could do it. Just that simple reinforcement gave me the confidence to pursue academia and research as a profession. I will never forget people like Amy Herring (Statistical Science) encouraging me to just “go for it”. The second reason why I believe that I was able to secure a tenure track position is because people made the interview process transparent. I like to say that sometimes academia can seem like a black box: people enter as graduate students or postdocs and then pop out as tenure track faculty without us really understanding the ins and outs of that evolutionary process. When I was submitting applications and going through interviews, faculty members outside of my immediate department around Duke really made sure that I was prepared. People like Jenny Tung (Biology), Tim Reddy (Biostatistics & Bioinformatics), and others took the time out of their busy schedules to coach me on what components they felt made up a good job talk versus what I needed to do to give a compelling chalk talk. Other faculty members took the time to read and edited my research and teaching statements. All those insights were so important because they allowed me to not have to walk into the job market blind.

Knowledge is just information that is meant to be shared. So, I would encourage early career scientists to build a diverse network of mentors and seek out additional guidance from those around them. I would also challenge senior scientists and faculty to normalize transparency in academia.

You have accomplished a lot very early, being included in the Top 100 most influential African Americans in 2019 among other honours. Do you have a message for the young scientists, particularly of minority ethnic groups worldwide?

Thanks so much for the kind words. I have been very fortunate. To be honest, those recognitions are something I could have never imagined happening to me. I struggled mightily during my first year in grad school and, quite frankly, there were instances where I seriously doubted if I was good enough to even be in a PhD program. The absolute best advice I ever received was to first ask myself if I belonged, simply answer “yes”, and then to never question myself again. So, to all young scientist, and especially to those of minority ethnic groups, yes there are aspects of research that are really difficult and sometimes academia can be seemingly full of rejection, but you are good enough to do this and your representation absolutely matters. Science needs you and you have a place here. What I would try to remember is that your career is not a race and solidifying your own independent research program takes time. Continue to chip away at it and don’t forget to continue to develop those intangible skills that make you unique.

What is the most exciting application that machine learning has to offer, in your opinion?

At the risk of sounding very biased, I think this is truly an awesome time to be working at the intersection of machine learning, biology, and health. As sequencing and imaging technologies continue to improve, there is a growing need for novel computational tools that better analyze the biological data that is being produced. With increasing sample sizes in these studies, there are a myriad of opportunities for machine learning techniques to be used in areas such as genomics, proteomics, and clinical informatics—and that’s not to mention all the new exciting multimodal approaches people have been taking recently to solve biomedical problems.

To me, the one application that I am most excited about is using machine learning to identify proximal or latent representations to genomic/clinical data that is generally difficult to collect in practice. Part of our motivation for wanting to understand the connection between shape and molecular activity is because taking multiple biopsies from patients can be challenging. Using machine learning to uncover unique fingerprints that connect shape morphology to disease mechanism might provide a path forward for the development of more noninvasive strategies that also lead to informed clinical diagnoses.

*This interview was conducted by Associate Editor Anam Akhtar*.

